# Neuropsychological and Behavioral Profile in Sleep-Related Hypermotor Epilepsy (SHE) and Disorders of Arousal (DOA): A Multimodal Analysis

**DOI:** 10.3390/jcm12010374

**Published:** 2023-01-03

**Authors:** Monica Puligheddu, Patrizia Congiu, Michela Figorilli, Ludovica Tamburrino, Patrizia Pisanu, Roberta Coa, Maria Giuseppina Mascia, Davide Fonti, Rosamaria Lecca, Enzo Grossi, Antonella Gagliano

**Affiliations:** 1 Sleep Disorder Center, Department of Public Health & Clinical and Molecular Medicine, University of Cagliari, 09042 Cagliari, Italy; 2 UOC Riabilitazione Funzionale e Neuroriabilitazione, Mater Olbia Hospital, 07026 Olbia, Italy; 3 ASL Cagliari, 09100 Cagliari, Italy; 4 Autism Research Unit, Villa Santa Maria Foundation, 22038 Tavernerio, Italy; 5 Unità di Neuropsichiatria dell’infanzia e dell’adolescenza, Dipartimento di Scienze Biomediche, Università di Cagliari & Ospedale Pediatrico, 09100 Cagliari, Italy

**Keywords:** sleep, cognitive, NREM, arousal, DOA, SHE, NREM parasomnia

## Abstract

Study Objectives: Disorder of arousal (DOA) and sleep-related hypermotor epilepsy (SHE) are complex, often bizarre, involuntary sleep behaviors, whose differential diagnosis may be challenging because they share some clinical features, such as sleep fragmentation. Mounting evidence highlights the critical role of sleep in cognitive functions. Controversial findings are raised about the cognitive profile in SHE; however, no studies have investigated the cognitive profile in DOA. This study aimed to assess whether sleep instability affects cognitive functions in patients with SHE or DOA. Methods: This study analyzed 11 patients with DOA, 11 patients with SHE, and 22 healthy controls (HC). They underwent full-night video polysomnography (vPSG) and comprehensive neuropsychological and behavioral evaluation. Differences in the variables of interest among the SHE group, DOA group, and their respective control groups were evaluated. The auto-contractive map (auto-CM) system was used to evaluate the strength of association across the collected data. Results: The SHE group had reduced sleep efficiency and increased wake after sleep onset (WASO); both the SHE and DOA groups showed increased % of N2 and REM sleep compared to the HC group. Neuropsychological and behavioral evaluations showed a different cognitive profile in the SHE group with respect to the HC group. The auto-CM showed that Pittsburgh Sleep Quality Index (PSQI), Beck depression inventory (BDI), MWCST_PE, Epworth sleepiness scale (ESS), WASO, N1, and % REM were strictly correlated with SHE, whereas the SE and arousal index (AI) were strictly related to DOA. Conclusions: Patients with SHE and DOA present different cognitive and psychiatric profiles, with subtle and selective cognitive impairments only in those with SHE, supporting the discriminative power of cognitive and psychiatric assessment in these two conditions.

## 1. Introduction

Disorders of arousal (DOA) and sleep-related hypermotor epilepsy (SHE) are complex, often bizarre, and sometimes violent involuntary behaviors occurring during sleep, whose differential diagnosis may be challenging because they share some clinical features.

DOA are non-rapid eye movement (NREM) sleep parasomnia embracing a spectrum of manifestation of increasing complexity, apparently focused and goal-directed, that occur as incomplete arousals from sleep [1]. DOA episodes might be associated with vegetative symptoms, automatic behaviors, misperception, and mental confusion. DOA usually appear in childhood, tend to disappear during adolescence, but might persist or emerge into adulthood [2]. Adults with DOA frequently complain of excessive daytime sleepiness, reduced daytime performances, and potentially harmful behaviors [3]. Frequently, DOA in adults show a fluctuating course, alternating periods of high frequency and intensity episodes with free intervals [3].

SHE is an epileptic syndrome characterized by stereotyped hypermotor seizures, occurring mainly during sleep, more precisely NREM sleep [4]. SHE-associated seizures are usually brief, lasting <2 min, are characterized by vigorous hyperkinetic behaviors with complex body movements, such as kicking and body rocking, and are frequently associated with vocalization, emotional grimaces, and autonomic signs [5,6]. The motor manifestations of SHE might be multifaceted, ranging from minor motor events and paroxysmal arousal recurring through the night to dystonic postures, complex hypermotor behavior, up to rarely epileptic nocturnal wandering [4,6]. SHE affects both sexes, with a peak of onset in childhood and adolescence, and might have a very high seizure frequency, occurring up to every night and usually many times per night [4,7]. Thus, patients with SHE frequently report fragmented sleep and daytime fatigue.

Sometimes, epileptic motor behaviors might be difficult to differentiate from parasomnias, and often, the ictal or interictal scalp electroencephalography (EEG) for SHE is not helpful, making the differential diagnosis with DOA challenging. Specifically, distinguishing seizures with paroxysmal arousals (SPAs) from simple arousal movements (SAMs) may be very difficult; thus, Loddo et al. [8] attempted to identify and better characterize the vPSG features of SPAs and SAMs. Moreover, neurological examination and neuroimaging are often normal in both conditions. To facilitate differential diagnosis between SHE and DOA in adults, Montini et al. [9] proposed a simple three-step diagnostic algorithm consisting of (1) clinical interview and physical examination, (2) questionnaires and home-made videos, and (3) vPSG.

Despite being two distinct clinical entities, DOA and SHE share fragmented sleep as an epiphenomenon, with important repercussions on daytime performance and an increased sense of daytime fatigue. Significant perturbation of sleep microstructures, through arousal-related phasic events and increased sleep instability, has been reported in patients with SHE and DOA [10,11,12]. Moreover, the two conditions may coexist in the same patient.

Increasing evidence has highlighted the critical role of sleep in cognitive functioning and consolidation [13,14,15,16]. Indeed, sleep stability and slow-wave activity have been linked to memory consolidation and improved cognitive performances [16,17].

Controversial findings are reported about the cognitive profile of patients with SHE, and some studies have reported deficits in extrafrontal and selective frontal cognitive functions, whereas others did not find any cognitive disturbances, even if chronically disrupted sleep and daytime sleepiness were noted in the majority of patients with sporadic SHE [18,19]

Conversely, no studies have examined the neuropsychological profile of patients with DOA.

To date, to the best of our knowledge, this study will be the first to assess the effect of sleep instability on the cognitive functions of patients with SHE and DOA.

This study aimed to assess, with a multimodal analysis, whether sleep instability in patients with SHE and DOA affects cognitive performances.

## 2. Materials and Methods

In total, 22 patients composed of 11 with DOA (3 men, mean age 32.3 ± 10.7 years) and 11 with SHE (5 men, 33.5 ± 15.2 years) were consecutively enrolled at the Sleep and Epilepsy Center of the University of Cagliari between 2015 and 2018, together with 22 age- and sex-matched healthy controls (HCs) (8 men, 30.5 ± 7.5 years). All patients were drug-free. SHE patients had a relatively mild disease.

For the DOA group, the inclusion criterion was having received the diagnosis of NREM parasomnia according to the current diagnostic criteria published in the International Classification of Sleep Disorders—3rd edition [1]. For the SHE group, the inclusion criterion was having received a diagnosis based on the current diagnostic criteria [4]. For both the DOA and SHE group, those with other sleep disorders, neurological disease, and/or psychiatric comorbidities according to the Diagnostic Statistical Manual V (DSM-V) were excluded [1,20]

Demographic and clinical data, such as age, sex, DOA duration, SHE duration, family history of DOA and/or SHE, and current therapy, were evaluated by expert neurologists in sleep medicine and epilepsy (MP and CP). Moreover, excessive daytime sleepiness was evaluated using the Epworth sleepiness scale (ESS) [21]. Finally, other sleep disorders were ruled out through a sleep-focused interview using validated questionnaires, namely, the Morning Evening Questionnaire [22] to assess circadian rhythm disorders and the Pittsburgh Sleep Quality Index (PSQI) [23] to estimate subjective sleep quality.

The local ethical committee approved the study (PROT. PG/2020/21657) and all participants gave written informed consent. The study was conducted according to the Declaration of Helsinki.

### 2.1. Neuropsychological Evaluation

All participants underwent a comprehensive neuropsychological assessment, including cognitive and behavioral evaluations, which was administered by a neuropsychologist according to standard procedures.

Global cognitive functions were assessed using the mini-mental state examination (MMSE) [24]. The neuropsychological battery included tests of long-term verbal episodic memory (immediate and delayed recall of a 15-word list) [25], long-term visuospatial episodic memory (Rey–Osterrieth complex figure test) [25], constructional praxis and visuospatial abilities (Rey’s figure copy), executive function by cognitive flexibility and nonverbal reasoning (modified card sorting test), selective attention, inhibition and processing speed (Stroop test) [26], selective attention and visual scan reactivity (visual search test) [27], visuospatial abilities and visual selective attention (trail-making test) [28], logical reasoning (Raven’s colored progressive matrices test) [29], short-term verbal memory (digit span forward test) [30], working memory (digit span backward) [31], and executive function and lexical access (phonological [25] and semantic verbal fluency [32]).

The behavioral evaluation included the assessment of depression using the Beck depression inventory (BDI) [33] and anxiety by the state–trait anxiety inventory [34].

### 2.2. Video Polysomnography (vPSG) Analysis

All participants underwent a full-night-attended vPSG recording in the sleep laboratory according to the American Academy of Sleep Medicine (AASM) recommendations [35] using Morpheus MICROMED ^®^ recorder and SystemPlus Evolution for data acquisition and scoring.

The vPSG montage included EEG leads placed following the 10–20 international system (Fp2, F4, F8, C4, P4, T4, T6, O2, Fz, Cz, Pz, Fp1, F3, F7, C3, P3, T3, T5, O1, referred to A1 or A2), left and right electrooculography, electromyography of the chin and lower limbs (tibialis anterior muscles), electrocardiography, nasal airflow, thoracic and abdominal respiratory effort, pulse oximetry, and microphone. The sampling rate was 256 Hz for all channels. To better observe any motor activity, all participants were asked to sleep uncovered and were provided with a light sheet for comfort.

The vPSG recordings were analyzed according to the AASM criteria [35], and the following sleep data were collected: total bed time, total sleep time, sleep efficiency, wake after sleep onset (WASO), percentage of time in each sleep stage (N1, N2, N3, and R), number of REM sleep episodes, arousal index (AI), periodic limb movement index (PLM index), and apnea–hypopnea index.

To detect minor and major events, all video recordings were carefully analyzed by experts in epilepsy and sleep medicine. In the SHE group, minor events were defined as nose scratching, dystonic posture of feet or hands, limb hyperextension, rigid posture of the upper or lower limbs, myoclonus, trunk flexion/extension, paroxysmal arousal, nocturnal wandering, and automatisms according to the current diagnostic criteria [4]. In patients with NREM parasomnias, simple and rising arousal movements were identified as minor events, according to the latest classification [36]. Major events were defined as complex hypermotor seizures in the SHE group [4] and as complex arousals with motor behaviors and walking movements in the NREM parasomnia group [36].

### 2.3. Statistical Analysis

#### 2.3.1. Classical statistical Analysis

The differences in variables of interest among the SHE group, DOA group, and their respective HC group were evaluated. After testing for distribution, the Mann–Whitney U test was used to evaluate differences in demographic, clinical, neuropsychological, and behavioral variables. Differences in PSG scores were evaluated using Student’s t-test. Pearson’s chi-squared test was performed to test differences in the distribution of nominal variables.

#### 2.3.2. Artificial Neural Network (ANN) Analysis

For the analysis of collected data, the auto-contractive map (auto-CM) system was used. ANNs are computational adaptive systems inspired by the functioning processes of the human brain; they are considered useful to solve nonlinear problems and discover subtle trends and associations among variables. Based on their learning through an adaptive way (i.e., extracting from the available data, the information needed to achieve a specific aim and generalize the acquired knowledge), the ANNs appear to be a powerful tool for data analysis in relatively small samples [37,38]. The auto-CM system is a fourth-generation unsupervised ANN, which has already been demonstrated to outperform several other unsupervised algorithms in a heterogeneous class of tasks [38,39]. Auto-CM was developed to explore the concomitant associations of different variables and the potential relationships among the same variables in a multi-factor network relevant for the disease. Once an auto-CM weights matrix is obtained, it is then filtered by a minimum spanning tree algorithm (MST) generating a graph. The goal of this data mining model is to discover hidden trends and associations among variables, since this algorithm is able to create a semantic connectivity map in which no linear associations are preserved, and explicit connection schemes are described. This approach shows the map of relevant connections between and among variables and the principal hubs of the system. Hubs can be defined as variables with the maximum number of connections in the map and the distances among variables reflect their bonding strength (weights). In other words, the auto-CM ‘spatializes’ the correlation among the variables (‘closeness’) and the graph identifies only the relevant associations, organizing them into a coherent picture. The Maximally Regular Graph (MRG) hubness function attains the highest value among all the graphs generated by adding back to the original MST, one by one, the missing connections previously skipped during the computation of the MST itself. In other words, the MRG generates, starting from the MST, the graph presenting the highest number of regular microstructures highlighting the most important connections of the dataset. To identify possible variables that have major effects on the results of the study, the first automatic analysis selected 25 primary items included in the auto-CM ANNs.

#### 2.3.3. Ethical Approval

This study was conducted in accordance with the Declaration of Helsinki, and the Independent Ethical Committee of Cagliari University Hospital approved the study (NP). All the parents received a full explanation of the study methods and purposes, and they gave their written consent.

## 3. Results

From a total of 120 consecutive outpatients referred for non-REM sleep motor disorders, 65 were diagnosed with SHE and 55 with DOA. Thus, in accordance with the above-mentioned inclusion and exclusion criteria, 22 patients were enrolled in this study. The demographic, clinical, neuropsychological, and behavioral data of the SHE and DOA groups, P values of the mean differences between the groups, and characteristics of the HC are shown in Table 1.

PSG features of the three groups are shown in Table 2.

The classical statistical analysis (p value of the statistical significance of the observed difference) showed some significant differences between the two groups (SHE and DOA) vs HC in PSG features (Table 2). Specifically, the SHE group showed a lower SE with respect to both the DOA (*p* = 0.01) and HC (*p* = 0.001) groups; no other differences in PSG features were found between the SHE group and the DOA group. Moreover, compared with the HC group, the SHE group showed higher WASO (*p* = 0.017) and both the SHE and DOA groups showed a significantly higher percentage of N2 sleep stage (*p* = 0.01; *p* = 0.005, respectively), a lower % of REM sleep (*p* = 0.005; *p* = 0.004, respectively), and a higher PLM index (although still within the normal range; *p* = 0.03; *p* = 0.009, respectively).

Noteworthily, the subjective sleep quality (PSQI) was significantly different between the SHE group and the DOA group (*p* < 0.002) and between the SHE group and the HC group (*p* < 0.001) (Table 1).

Among the cognitive parameters, short-term memory (digit span forward) and verbal learning are the only two variables that differentiate the two clinical groups (*p* < 0.008 and *p* < 0.01, respectively). The cognitive parameters were not different between the DOA group and the HC group. On the contrary, the working memory (digit span backward) (*p* < 0.03) and semantic fluency (*p* < 0.01) were different between the SHE group and the HC group.

Only the SHE group appears to suffer from depression, as suggested by the significant difference in the BDI score between the SHE group and the HC group. Consequently, the mean scores on BDI are significantly different (*p* < 0.013) between the SHE group and the DOA group.

**Figure 1:**
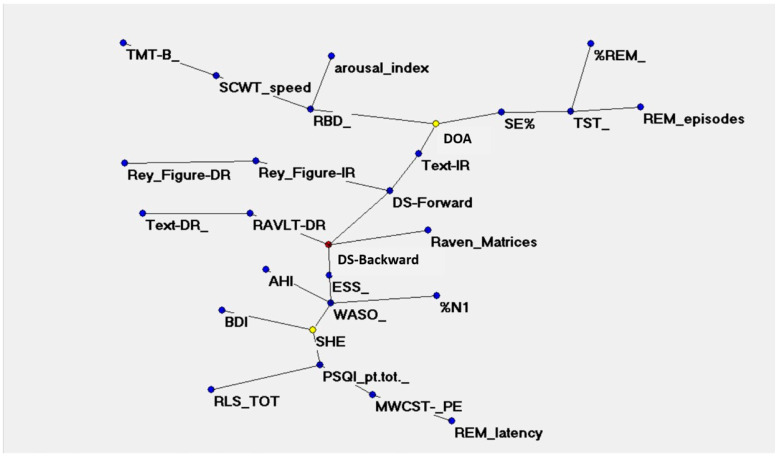
**The figure shows the connections between the sleep and the cognitive parameters. Abbreviations:** AHI: apnea-hypopnea index; BDI: Beck Depression Inventory; DOA: disorders of arousal; DR: delayed recall; DS: digit span; ESS: Epworth Sleepiness Scale; IR: immediate recall; MWCST: modified Wisconsin card sorting test; %N1: percentage of N1 sleep stage in total sleep time; PSQI_pt-tot.: total score of Pittsburgh sleep questionnaire index; RAVLT; Rey auditory–verbal learning test; RBD: REM sleep behavior disorder; %REM: percentage of REM sleep in total sleep time; RLS: restless legs syndrome; REM: rapid eyes movement sleep stage; SCWT speed: The Stroop Color and Word Test; SE%: sleep efficiency; SHE: Sleep hypermotor epilepsy; TMT-B: trail making test – B; TST: total sleep time; WASO: wakefulness after sleep onset. Yellow dots are referred to DOA and SHE; blue dots are referred to sleep and neuropsychological features.

The semantic connectivity map (auto-CM method)–MSTgraph (Figure 1) showed the strength of the association across the clinical and PSG variables visualized by the concept of “closeness”: the variables with higher-connection weights are relatively nearer and vice versa.

As shown in the figure, the “maximally regular graph” superimposed to MST indicates that both DOA and SHE are linked to several sleep and cognitive parameters and that many of these parameters are shared between DOA and SHE. The links’ strength values of the parameters with other variables included in the graph were all very high (‡0.8), indicating a strong probability of co-occurrence of these symptoms and signs. Specifically, PSQI, BDI, and MWCST_PE, as behavioral parameters, and the ESS, WASO, N1, and % REM, as sleep parameters, strictly correlated with SHE. On the contrary, the SE and AI are strictly related to DOA.

## 4. Discussion

This study describes the neuropsychological profiles of patients affected by sleep hypermotor epilepsy (SHE) and by disorders of arousal (DOA), identifying slight but discriminatory cognitive differences between the two groups. Furthermore, it reports a set of sleep parameters recorded by vPSG (namely, SE, WASO, percentage of N2 sleep stage) that may be useful diagnostic supporting tools for discriminating between SHE and DOA.

Previous studies have provided numerous data on the sleep characteristics of both conditions, but very few clinical parameters appeared useful in distinguishing between them without vPSG [40].

A recent study [41] showed a slight but significant difference between the DOA and SHE groups in sleep characteristics, such as sleep efficiency, light sleep, deep sleep, REM sleep, CAP subtypes, SWS fragmentation, and representation of stage N3, and reported that the only discriminating elements between the two conditions were sleep length (more reduced in DOA) and sleep instability (more elevated in SHE). The authors conclude the existence of an underestimated continuum across the two conditions, linked by increased levels of sleep instability and higher rates of slow-wave sleep and NREM/REM sleep imbalance. Recently, to facilitate the differential diagnosis between SHE and DOA in adults, Montini et al. [9] proposed a simple diagnostic algorithm consisting of three steps: (1) clinical interview and physical examination, (2) questionnaires and home-made videos, and (3) video PSG.

This study assessed the cognitive effect of sleep instability in the SHE and DOA groups and showed discriminatory neuropsychological features, suggesting that the strongest differences between the two conditions could be detected in the cognitive profiles.

Among the neuropsychological variables, we identified two cognitive parameters that appear to be affected in patients with SHE and preserved in those with DOA: short-term memory (assessed by the digit span forward test) and verbal learning (assessed by the auditory–verbal learning test (RAVLT)). The digit forward span test captures short-term memory abilities and attention efficiency and capacity. Some authors inferred that memory deficit may be linked to the direct negative effect of interictal and ictal frontal or extrafrontal epileptic activity and by the alterations in sleep-related encoding memory processes induced by sleep fragmentation [42,43]. On the contrary, sleep deprivation exerts a negative effect on cognitive functions, including memory retention and several domains of executive functions [44]. The RAVLT aims to assess the immediate episodic declarative memory and new verbal learning (retention of information after a period and memory recognition) [45] and is a sensitive tool to reveal neurological impairment and verbal memory deficits in various patient groups, including those suffering from left-temporal epilepsy [46]. Scores of verbal learning and verbal episodic declarative memory on the RAVLT are also strongly related to executive function [47].

The original assumption that patients with SHE do not report gross psychological and cognitive deficits [48] was questioned by some studies, revealing intellectual disability and psychiatric problems in patients with SHE carrying nAChR subunits and *KCNT1* gene mutations [49]. None of our patients showed global impairment in cognitive function. Our data are inconsistent with a more recent report [50], revealing intellectual disabilities or a borderline IQ in 12% of the patients with SHE.

Compared with the HC group, the SHE group displayed subtle neuropsychological impairments concerning working memory, as revealed by the digit span backward test, an executive task dependent on working memory, and in semantic fluency, as revealed by a semantic verbal fluency task. Our results are consistent with previous studies showing deficits in memory functions and executive working memory in cohorts of patients with sporadic and familial SHE, irrespective of the treatment status [19,51,52].

Impairments in semantic fluency may be related to a deficiency in sustained retrieval management or to a failure of semantic/conceptual memory. It has been seen as pointing to either frontal or temporal dysfunctions of the language-dominant hemisphere [53]. A primary study found both semantic and phonemic fluency deficits in temporal lobe epilepsy [54].

Meaningfully, the cognitive parameters were not different between the DOA group and the HC group. This result underlines the difference in cognitive features between SHE and DOA. It is related to the lack of evidence on cognitive impairment in DOA.

Finally, only the SHE group appears to experience depression, as revealed by the BDI. Some authors suggested that sleep deprivation may negatively affect cognitive systems connected to emotional networks [44]. A previous study [50] reported psychiatric disorders, such as anxiety and depression, in 24% of patients with SHE. On the contrary, no previous studies of psychiatric disorders in patients with DOA are available in the literature. Consistently, we did not find depressive symptoms in our patients with DOA. Thus, we suggest considering the role of psychiatric evaluation in the differential diagnosis between SHE and DOA.

### Semantic Network Analysis

To the best of our knowledge, this is the first study to have adopted a complex network mathematics approach, such as auto-CM, to face the complexity of DOA and SHE conditions. The semantic connectivity map shows the lack of a clear segregation between DOA and SHE and allows for grasping the core of the relationship between both sleep and cognitive variables of the two conditions. Exploiting all not-obvious connections among the full spectrum of the variables reveals a substantial overlap between DOA and SHE because some cognitive parameters, such as MMSE and Rey copy, appear to represent shared hubs between SHE and DOA.

Although the classical statistical analysis showed some substantial differences between the two clinical groups, the results were far less explicative of the whole relationships between the variables. Only by displaying the hidden links between variables, the complexity of the clinical expressions of both DOA and SHE can be represented, as the two conditions affect both sleep and cognitive domains.

## 5. Limitations

First, the study had a small sample size. However, all enrolled patients were highly selected, drug-free at the time of video PSG recordings and neuropsychological evaluation, and had no other comorbidity. Second, the enrolled patients had relatively mild disease severity. This could explain the lack of individuals with intellectual disabilities among our patients. According to the results of the study by Licchetta et al. [19], variables of clinical severity (i.e., high seizure frequency, status epilepticus, bilateral convulsive seizures, and poor response to antiepileptic treatment) were negatively correlated with memory and executive functions. Third, our study did not provide the genetic profile of the patients. Therefore, it cannot present a correlation between the neuropsychological profile and putative gene mutations, such as the nAChR subunits and *KCNT1* genes, that are supposed to be responsible for a more severe presentation of SHE [50]. Indeed, Licchetta et al. [19] found that patients with mutated SHE, compared to nonmutated ones, irrespective of the specific gene, presented a lower IQ. However, our patients with SHE showed a clear specific neuropsychological impairment, despite the mild disease presentation.

## 6. Conclusions

This study describes different neuropsychological and psychiatric profiles between patients with SHE and DOA, revealing subtle and selective cognitive impairments in different aspects of executive functions, attention, and memory, only in patients with SHE when compared with HC, as well as depression only in patients with SHE. The substantial integrity of the cognitive skills and mood of patients with DOA supports the discriminative power of cognitive and psychiatric assessment in these two conditions.

If future studies, including more participants, can confirm our data, the neuropsychological and mood profiles can be considered a noteworthy discriminant factor between the two conditions and raise a suggestion in understanding the peculiarities of the clinical features of SHE and DOA.

## Figures and Tables

**Table 1:** Demographic, clinical, neuropsychological, and behavioral data of the SHE group, DOA group, and healthy control group.

	SHE (11)	DOA (11)	*p* Value	SHE (11)	HC (11)	*p* Value	DOA (11)	HC (11)	*p* Value
Age	25.9 (18.8–51.8)	32.8 (22.7–38.5)	0.95	25.9 (18.8–51.8)	29.0 (25.0–49.5)	0.61	32.8 (22.7–38.5)	34.0 (25.0–39.0)	0.90
Gender (M)	5\11	3\11	0.4	5\11	5\11	1.0	3\11	3\11	1.0
Subjective sleep quality (PSQI)	9,0 (9.9–13.0)	7.0 (5.0–8.0)	0.002	9.0 (9.9–13.0)	4.0 (2.2–4.0)	<0.001	7.0 (5.0–8.0)	5.0 (5.0–6.0)	0.10
Excessive daytime sleepiness (ESS)	7.0 (3.0–9.0)	6.0 (5.0–8.0)	0.95	7.0 (3.0–9.0)	5.5 (2.5–8.0)	0.69	6.0 (5.0–8.0)	9.0 (2.0–9.0)	0.65
Global cognitive function (MMSE)	27.9 (26.6–30.0)	28.2 (27.6–30.0)	0.65	27.9 (26.6–30.0)	28.4 (27.4–30.0)	0.52	28.2 (27.6–30.0)	30.0 (27.1–30.0)	0.85
Long-term verbal memory (15-words Rey)	7.0 (5.4–8.5)	6.2 (5.6–9.6)	0.80	7.0 (5.4–8.5)	7.9 (7.0–8.9)	0.21	6.2 (5.6–9.6)	7.6 (6.4–8.8)	0.56
Visuo-constructional functions (Rey figure)	10.6 (5.2–16.3)	17.0 (6.0–18.0)	0.52	10.6 (5.2–16.3)	13.4 (10.8–18.9)	0.19	17.0 (6.0–18.0)	13.1 (9.5–19.1)	0.80
Short-term memory (Digit span forward)	4.5 (4.0–5.5)	5.5 (5.2–6.5)	0.008	4.5 (4.0–5.5)	4.2 (3.6–4.7)	0.29	5.5 (5.2–6.5)	5.0 (4.4–5.5)	0.03
Working memory (Digit span backward)	4.0 (4.0–4.0)	4.0 (4.0–5.0)	0.56	4.0 (4.0–4.0)	5.0 (4.2–5.0)	0.03	4.0 (4.0–5.0)	5.0 (4.0–5.0)	0.17
Non-verbal reasoning and cognitive flexibility (MWCST)	1.1 (0.0–6.2)	1.3 (0.0–2.8)	0.95	1.1 (0.0–6.2)	0.0 (0.0–1.1)	0.23	1.3 (0.0–2.8)	1.3 (0.0–1.8)	0.95
Inhibition and selective attention (Stroop)	0.0 (0.0–0.2)	1.7 (0.0–3.0)	0.13	0.0 (0.0–0.2)	0.0 (0.0–0.0)	0.74	1.7 (0.0–3.0)	1.5 (0.0–2.25)	0.52
Selective attention and visual scan reactivity (visual search)	46.2 (39.7–52.7)	47.0 (44.2–49.7)	0.90	46.2 (39.7–52.7)	48.7 (43.6–51.5)	0.83	47.0 (44.2–49.7)	44.7 (42.2–47.2)	0.44
Verbal learning	4.4 ± 1.1	5.8 ± 0.7	<0.01	4.4 ± 1.1	4.5 ± 0.8	0.95	5.8 ± 0.7	5.6 ± 1.1	0.61
Visuospatial abilities (TMT)	113.0 (96.0–134.0)	104.0 (80.0–133.0)	0.52	113.0 (96.0–134.0)	90.5 (73.0–122.2)	0.91	104.0 (80.0–133.0)	116.0 (65.0–131.0)	1.00
Phonemic fluency	23.2 (17.5–29.5)	27.3 (19.0–31.1)	0.75	23.2 (17.5–29.5)	28.7 (25.6–33.8)	0.17	27.3 (19.0–31.1)	26.1 (19.6–29.3)	0.70
Semantic fluency	35.0 (28.0–41.0)	34.0 (31.0–41.0)	1.00	35.0 (28.0–41.0)	42.0 (37.5–51.0)	0.01	34.0 (31.0–41.0)	36.0 (31.0–39.0)	0.90
Abstract reasoning (MP47)	29.0 (23.0–31.5)	27.5 (26.5–31.0)	0.70	29.0 (23.0–31.5)	32.5 (28.9–35.4)	0.08	27.5 (26.5–31.0)	31.0 (28.5–36.0)	0.19
Depression (BDI)	10.0 (8.0–14.0)	5.0 (4.0–7.0)	0.013	10.0 (8.0–14.0)	3.0 (0.0–4.7)	<0.001	5.0 (4.0–7.0)	3.0 (0.0–10.0)	0.75
Anxiety (STAI-Y)	42.0 (41.0–48.0)	42.0 (39.0–48.0)	0.85	42.0 (41.0–48.0)	41.0 (38.0–42.7)	0.23	42.0 (39.0–48.0)	44.0 (41.0–48.0)	0.52

BDI, Beck Depression Inventory; DOA, disorders of arousal; HC, healthy controls; MMSE, mini-mental state examination; MP47, Raven’s colored progressive matrices test; MWCST, modified Wisconsin card sorting test; STAI-Y, State–trait anxiety inventory Y; TMT, trail-making test. Data are expressed as median and interquartile range.

**Table 2:** Polysomnography features of the SHE group, DOA group, and healthy control group.

	SHE	DOA	*p* Value	SHE	HC	*p* Value	DOA	HC	*p* Value
	N (11)	N (11)		N (11)	N (11)		N (11)	N (11)	
TST	413.7 ± 65.2	467.5 ± 41.1	0.03 *	413.7 ± 65.2	441.3 ± 43.2	0.27	467.5 ± 41.1	455.2 ± 52.5	0.58
SE	79.5 ± 9.8	89.8 ± 6.2	0.01 *	79.5 ± 9.8	93.3 ± 5.5	0.001 *	89.8 ± 6.2	92.4 ± 5.3	0.31
Sleep latency	27.5 ± 24.4	14.3 ± 12.0	0.13	27.5 ± 24.4	4.5 ± 2.1	0.11	14.3 ± 12.0	6.5 ± 5.3	0.07
Arousal Index	8.4 ± 4.0	9.8 ± 7.0	0.58	8.4 ± 4.0	9.2 ± 4.1	0.7	9.8 ± 7.0	6.7 ± 2.4	0.2
WASO	63.4 ± 50.5	32.7 ± 27.2	0.1	63.4 ± 50.5	18.5 ±17.2	0.017 *	32.7 ± 27.2	21.4 ± 18.0	0.28
N1	9.4 ± 6.3	6.0 ± 2.1	0.12	9.4 ± 6.3	8.2 ± 4.7	0.62	6.0 ± 2.1	8.0 ± 4.7	0.27
N2	48.1 ± 16.7	42.2 ± 7.5	0.3	48.1 ± 16.7	31.7 ± 3.8	0.01 *	42.2 ± 7.5	33.5 ± 4.4	0.005 *
N3	29.5 ± 14.7	31.8 ± 11.7	0.68	29.5 ± 14.7	36.9 ± 4.5	0.14	31.8 ± 11.7	32.9 ± 5.6	0.79
REM	13.1 ± 8.6	17.4 ± 4.4	0.16	13.1 ± 8.6	23.2 ± 5.4	0.005 *	17.4 ± 4.4	25.5 ± 5.8	0.004 *
PLMS index	10.8 ± 13.9	5.8 ± 5.1	0.28	10.8 ± 13.9	0.46 ± 0.91	0.03 *	5.8 ± 5.1	0.6 ± 1.3	0.009 *
AHI	2.7 ± 6.1	0.6 ± 1.4	0.28	2.7 ± 6.1	0.16 ± 0.47	0.19	0.6 ± 1.4	0.2 ± 0.5	0.32
Minor motor events	57.3 ± 38.2	49.1 ± 22.8	0.55	57.3 ± 38.2	\	\	49.1 ± 22.8	\	\

AHI, apnea–hypopnea index; DOA, disorders of arousal; ESS, Epworth sleepiness scale; HC, healthy controls; TST, total sleep time; N1, % of NREM sleep stage 1 in TST; N2, % of NREM sleep stage 2 in TST; N3, % of NREM sleep stage 3 in TST; PLMS, periodic limb movements of sleep; PSQI, Pittsburg sleep quality index; REM, % of REM sleep in TST; SE, sleep efficiency; WASO, wake after sleep onset in minutes. Data are expressed as median and standard deviation. * is referred to statistically significant results (*p* ≤ 0.05).

## Data Availability

Data are available on request.
